# Sensibility—Neuroses of the Pharynx and Larynx

**Published:** 1881-09

**Authors:** A. Jurasz

**Affiliations:** Heidelberg


### SENSIBILITY—NEUROSES OF THE PHARYNX AND LARYNX.

By PROF. A. JURASZ, of Heidelberg.

Translated for the Medical and Surgical Reporter.

*Gentlemen.*—The patient whom I now introduce to you has been sent to me for the purpose of being subjected to a closer examination. He is a laborer, aged 35 years, very well nourished and strongly built, and says that he has never been seriously ill.

Two days ago, while drinking with a companion, he quarreled with the latter, and at the moment when he had the glass at his lips, received a severe blow from him, so that the glass was broken into small fragments. Now he maintains that he swallowed a piece of the glass, and complains of a continuous pain in his throat, especially when he attempts to swallow.

Upon closer questioning we learn that the pain is referred to the pharynx near the larynx, and that it was more severe yesterday than it is to-day.

Professor Braun examined the patient yesterday, but neither by palpitation nor inspection was he able to discover any foreign body, and he is before us now for the purpose of submitting to an examination with the laryngoscope, so that we may discover, if possible, the cause of his suffering.

Upon examination we find the mucous membrane of the pharynx of normal color; neither do we find any anomaly when we examine the posterior nares, by means of the rhinoscope. A foreign body, or a lesion produced by one, is nowhere discoverable. The larynx is also perfectly intact, phonation is normally produced and the voice is clear. When I touch the walls of the pharynx with a laryngeal sound, we find one point which, when touched, gives the patient pain. This point is situated on the posterior wall of the pharynx, about opposite the middle of the upright epiglottis. The mucous membrane in this region is perfectly normal. By moving the sound about over the mucous membrane, we are enabled to say with certainty that neither here nor anywhere else is there imbedded a foreign body. And yet the patient is perfectly sure that there is a piece of glass in his throat. We must, therefore, ask ourselves the question, Have we here a case in which a painful sensation is produced without any discoverable cause, or is the patient simulating?

The latter is not very probable, because the patient declares he has no ill will against his friend, and that he does not intend to take any legal steps against him. We have, therefore, no cause to doubt his veracity.

But, if his assertions are plausible, we must then decide whether he has really swallowed a piece of the broken glass or not, and if the first, whether the fragment is not somewhere imbedded in the tissues, or whether, if already removed, the pain is owing only to the injury.

The first question we have already answered, by our examination, in the negative. Yet I must here state that we must in such cases make a very minute examination of all the parts before we state positively that there is no foreign body present, as such a body may be far removed from the spot where the patient apparently feels it. This is owing to the poorly developed sense of touch of the mucous membrane of this region.

The second question refers to the possibility that a fragment of glass may have been present for a time, and that after having been removed, the pain may be caused by the injury inflicted. But this, also, is not the case; neither Prof. Braun nor we have been able to find even the slightest swelling or hyperæmia.

Therefore, we are led to assert that our patient is mistaken when he believes there is a fragment of glass imbeded in his throat, or that there has ever been one there. The pains of which he complains are the expression of a disturbance of the tactile sensation, which we can explain about as follows: At the moment of the accident our patient was very much excited. The possibility of his having swallowed a fragment of glass became, in consequence of the great fear of most people of such an occurrence, to him, a certainty. From this to feeling the body, was, owing to his excited condition, only a step, and thus we find him an excellent example of a phantastic paræthesia of the pharynx, such as is not seldom met with in practice.

The pathogeny of this case is not without interest, as it belongs to a class of cases which are met with often enough in general practice, but which have, until now, seldom been the subject of scientific research. These are the anomalies of sensibility of the pharynx and larynx. They are divisible into two groups: The anomalies of *kind* and *quality of perception*, or feeling, and anomalies of *quantity* or *force* of perception. The first are termed paræthesias, and by that term we mean those forms of abnormal feeling which are either entirely of spontaneous origin, or are caused by very insignificant local irritations. These perceptions do not correspond with the local changes in any case.

The anomalies of quantity and force of perception, on the contrary, are relative to the latter, and are, if the sensibility is less than normal, called hypasthesia and anæsthesia, or if it is increased, hyperæsthesia and hyperalgesia.

Neuralgia of these organs is classed with neither of these forms, but is considered as a distinct form. Its chief symptom is the pain, which is paroxysmal and periodical and there are no local changes discoverable.

Paræsthesia of the pharynx and larynx makes itself manifest by a great variety of sensations; the patients complain of oppression, tickling, burning, dryness or soreness; in other cases they maintain that they feel something creeping about in their throat, or that a foreign body is imbedded therein.

Among the affections which are accompanied with a special predisposition to paræthesia of the throat, we must give hysteria the most prominent place. The so-called globus hystericus is described as a ball which ascends from the stomach and is impacted either in the pharnyx or larynx, and which often gives rise to the most intense dyspnœa. Other sensations, such as expiring cold air, straw, etc , in the throat, have been described.

Scarcely less seldom do we find paræsthesia of the throat connected with hypochondriasis. These patients imagine themselves suffering with laryngeal phthisis, and complain of pain, burning and soreness of the throat, of which they are not relieved until they notice some other trouble, which still more endangers their lives.

Popular treatise on diseases of these organs can often be proven as a cause for such troubles. All these abnormal sensations can subside in a short time, or they can continue for a long period. The latter is especially the case with speakers and singers.

Another affection which can be accompanied with paræsthesia of these organs is anæmia, and it also very often accompanies chronic inflammatory diseases of the lungs.

Curious and interesting are those paræsthesias of the throat which we meet with in perfectly healthy individuals when under the influence of some severe excitement, such as the patient whom we have just examined shows. It is a remarkable fact that this trouble is seldom met with in genuine local changes: inflammations and ulcerations run their course without producing relatively severe symptoms.

Lastly, paræsthesia of the throat sometimes accompanies the first stages of bulbar paralysis and paralysis of the sensible and motor tracts of the pharynx and larynx. These are accompanied by numbness, dryness and formication.

Hyperæsthesia of the pharynx and larynx is that increased irritability which is caused by demonstrable peripheral irritation. An irritation may, therefore, cause a common, but increased perception, which may be felt as pain or which may be accompanied only by reflex manifestations.

Hyperæsthesia of the throat can be present without any discoverable change of the organic structure. It is sometimes so excessive, that, not only when the mucous coat is touched, but even when it is only approached, an unpleasant sensation and severe reflex action (gagging, coughing, vomiting), are produced.

I have been unable to satisfy myself that this idiopathic hyperæsthesia is oftener met with in females than in males, as some inquirers maintain. I can, however, confirm the observation that it accompanies many physiological actions, such as dentition, menstruation and pregnancy. In such cases it developes itself with the baginning of these actions and disappears with their ending. Violent emotions are also able to produce hyperæsthesia. Most frequently hyperæsthesia of the throat and larynx is caused by the local affections of these organs. Catarrhal phlegmonous and erysipelatous affections are always accompanied by a more or less sensitive condition of these organs.

Ulcers and changes of a tuberculous nature are also sometimes accompanied by hyperæsthesia. On the contrary, it is generally missing in syphilitic ulcers and in neoplasms, benign as well as malignant.

Phthisis pulmonalis is also sometimes combined with an excessive hyperæsthesia of the pharynx and larynx, without any particular change of the mucous membrane of these parts. Neuralgia of these organs is very rare.

Anæsthesia of the pharynx and larynx is characterized either by a diminution of the normal sensibility, or by a loss of the preception of pain. The first form is not always positively a pathological one, because the sensibility may, under normal conditions, be very poorly developed. A safe criterion in such cases is the coexistence of moto nuroses, or some other affection of the nerves of these organs, with the anæsthesia. The symptoms of the severer forms of anæsthesia of these parts vary according to the nerves affected. Sensible paralyses of the recurrents cause the least inconvenience; those of the superior laryngeal nerves, however, give rise to serious trouble. Owing to the paralysis of the thyroid and aryepiglottic muscles the opening into the larynx is not properly closed during deglutition; at the same time the mucous membrane of the entire region is insensible, and so it happens that food is easily forced into the larynx, and through it into the bronchi, until it reaches a more sensitive part of the mucous membrane and gives rise to very severe paroxyms of coughing. If we examine the patient with the laryngoscope we will find the epiglottis upright and the mucous membrane somewhat reddened, owing, probably, to the repeated irritation produced by foreign substances. The membranes can be touched without producing any reflex action and without pain. The same condition is found in paralysis and anæsthesia of the pharynx. In these cases swallowing is often very difficult, or the food may be ejected through the nostrils. The anæsthesia may be uni- or bi-lateral, and it may be more complete on one side than on the other. The patients often complain of a sensation of formication, burning, tickling, etc., and even pain may be felt (anæsthesia dolorosa).

Anæsthesia of the pharynx and larynx is most frequently seen in connection with hysteria. Less complete but relatively often it is found with anæmia and chlorosis. As a constant symptom it is also found during epileptic paroxyms, and during the asphyctic stage of Asiatic cholera. It may also be produced by certain medicines; especially the narcotics, when applied either locally or given internally; also by bromide of potassium. It has long been known as a sequel of diphtheria; it is developed in from one to four weeks after recovering from this affection, and may be complete or incomplete, uni- or bilateral, and more or less circumscribed.

If now, gentlemen, we try to explain the pathogeny of these affections, we must remember that they are undoubtedly symptoms of an abnormal irritability. Such an irritability can be produced by a great variety of irritants.

For the sake of brevity I will call those irritations which are produced in the periphery, peripheral; and those which are of central origin, central.

Paræsthesia can be caused by an irritation of the central organs; this is the case in hypochondriasis. Here the irritation is entirely a psychical one, for we fail to find any change in the peripheral organs or their nerves. The simple idea which is transferred under the influence of the consciousness of a strong intention of the mind to the periphery is strong enough to produce the most variable sensations in the pharynx or larynx.

In this manner emotional excitement may give rise to paræsthesia. Here the intention of the mind is momentarily directed to these organs by some accident, and the simple idea is changed into an apparently real perception, which perception in turn again acts as an irritant, and changes the momentary disposition of the mind into a permanent one.

Whether the seat of the irritation is central in those paræsthesias which accompany anæmia and chlorosis cannot be positively stated, but this seems the most probable, as the deficient nutrition of the central nervous system can give rise to false perceptions in the pharynx and larynx, while, on the contrary, anæmia of these parts will not produce them,

So, too, these paræthesias which are referable to a very slight peripheral irritation may be classed as central, because the peripheral irritation may be only an opportune one, and the real source may be cęntral in origin. It is the fear which most persons have of swallowing pieces of bone, glass, etc., which causes them to convert a very slight irritation into the most extreme pain. On the contrary, the irritation is produced in the periphery when the paræthesia is the direct result of some local lesion which is proportional to the sensations produced. The irritation is peripheral which produces those paræthesias which accompany the beginning of destructive processes in the lungs.

Of the localization of the irritation in hysterical paræsthesia I can say nothing positive. The fact that hysteria may be produced by both peripheral and central disturbances, makes it probable that the origin of hysterical paræthesia of these organs may also be either central or peripheral.

The pathogeny of hyperæsthesia of the pharynx and larynx needs but a few words after what I have said of paræsthesia. In those cases in which it is idiopathic, it may be regarded as one of the symptoms of some general affection. It may also have a local cause, which, indeed, is not always demonstrable, but we have seen that this effect may be produced by a rapid succession of touches or by inflammations.

Anæsthesia is generally produced by a material change in the nervous apparatus of the parts. We must seek the cause in the spinal centres in those cases in which our affection is only a symptom of a cerebral or spinal disease· Anæmic anæsthesias are also probably of central origin. On the contrary, the anæsthesia which follows diphtheria seems to be of peripheral origin.

The prognosis of the paræsthesias is generally favorable, although it should generally be cautiously stated. The same can be said of hyperæsthesia, and also of anæsthesia, but an extreme degree of anæsthesia of the larynx may be dangerous, because foreign bodies may, at any time, enter the air passages and thus cause death.

The therapeutics of these affections must be directed against the affections which they accompany. Only when the primary illness is incurable, or when the sensibility neuroses are independent of other troubles, is it necessary to direct our efforts against them *per se*.

Paræsthesia may sometimes be subsided by having recourse to delusive measures with the object of producing a psychical effect.

Hyperæsthesia requires especially a local treatment. Astringents, in the form of gargles and inhalations, painting with solution of potassium bromide, hydrate of chloral and morphia, are beneficial.

Anæsthesia of the pharynx and larynx being generally self-limited, needs little treatment unless it becomes very complete; in such cases care must be taken that life is not endangered by the introduction of foreign bodies into the air passages. Food should, therefore, be given through an œsophageal tube, care being taken in its introduction, so that it does not enter the larynx.

Of internal remedies strychnia is especially beneficial. Electricity, in the form of the constant or induced current directly to the affected parts, is also beneficial.

